# Application of 2-Trichloromethylbenzimidazole in Analytical Chemistry: A Highly Selective Chromogenic Reagent for Thin-Layer Chromatography and Some Other Analytical Uses

**DOI:** 10.1155/2012/650150

**Published:** 2012-04-04

**Authors:** Leszek Konopski, Anna Kiełczewska

**Affiliations:** Analytical Department, Institute of Industrial Organic Chemistry, Annopol 6, 03-236 Warsaw, Poland

## Abstract

2-Trichloromethylbenzimidazole (TCMB) was used as a chromogenic reagent in organic or inorganic analysis, mainly in thin-layer chromatography (TLC). In reactions of TCMB with some heteroaromatic nitrogen containing compounds, such as azines, azoles and benzazoles, a formation of high colored products occurred. For azines, the chromogenic reaction was highly regioselective, since the both adjacent **α**-positions *versus* the nitrogen atom(s) must not be substituted. A TLC method of detection was developed. Thirty azines, azoles, and benzazoles were detected at the detection limit 10 ng to 1 **μ**g. This method was also applied for detection of heteroaromatic pesticides, and the attempts to construct active and passive dosimeters for nicotine were made. In a prechromatographic reaction of aromatic *o*-diamines with methyl trichloroacetimidate, TCMB or its derivatives were formed *in situ*. Followed by TLC and visualization in pyridine vapors, this procedure was applied for detection of *o*-phenylenediamine derivatives. The reaction product of TCMB and pyridine (LI Complex) was identified and fully characterized. Two different reaction mechanisms: with electron deficient basic heteroaromatic compounds, like pyridine, and with more acidic compounds, for example, pyrrole, were discussed. In aqueous solutions, the LI Complex may be also used as a new indicator for complexometric, adsorption and acid-base titration of inorganic compounds.

## 1. Introduction

The synthesis of 2-trichloromethylbenzimidazole (TCMB, **1**) was first described in 1967 by Holan and coworkers [1]. Some reactions of this highly reactive compound with different nucleophiles, such as liquid ammonia (with 2-cyanobenzimidazole formation, in a rather unusual transformation of –CCl_3_ group into –C*≡*N [2]), primary and secondary amines, bifunctional compounds [3], water, alcohols, and thiols [4] were studied by the same team. The only mention concerning reactions of the compound (**1**) with tertiary aliphatic amines was that some unstable and unidentified compounds are formed [2].

 TCMB was a key starting material during preparation the Ph.D. dissertation of Dr. Konopski in early 1970s. One of unsolved issues was purification of this compound, originally crystallized from high-boiling nitrobenzene, what was not convenient. Once during crystallization attempts trying various organic solvents it was unexpectedly found that colorless (**1**), usually sparingly soluble in most of solvents, did dissolved very easily and underwent a vigorous reaction with pyridine **2** at room temperature. During several seconds the colorless solution became yellow, green, blue, violet, and purple, consecutively, and after about 2 min of the exothermic reaction, an intense amaranth solid precipitated from the pyridine solution. The high-colored product (LI Complex) remained unidentified until 1980s. In this paper, a review of LI Complex and another high-colored product identification and application of this color reaction in several analytical methods for both organic and inorganic compounds has been presented.

## 2. TCMB as a High-Colored Chromogenic Agent for TLC of Heteroaromatic Compounds

### 2.1. Azines

The reactions of TCMB with different heteroaromatic compounds have been systematically studied by Konopski and coworkers since 1986. It was found that analogical colored products as with pyridine **2** were formed in the reactions of TCMB with some pyridine derivatives substituted at the 3- and/or 4-positions but with both 2- and 6-positions free [5]. Similar reactions with high-colored products formation were observed also for diazines, such as pyridazine **3**, pyrimidine **4**, and pyrazine **5** but always the necessary condition was that both *α*-positions versus at least one nitrogen atom in the azine ring should be unsubstituted [5]. Although the reaction occurred already at room temperature (RT), it was observed that it may be accelerated by heating to 100–150°C. This reaction was used for selective detection of some pyridine and diazines derivative unsubstituted in both *α*-positions on thin-layer chromatographic (TLC) plates [5]. Standard Merck aluminum plates coated with Silicagel (Merck) and a solvent system appropriate for the detected compounds were used. The developed plates were dried on air, sprayed with 1% TCMB acetone solution, and placed in a laboratory drier for 1 min at 150°C, giving intensively colored spots with a decent low limit of detection (LOD) of 10–100 ng (Table 1).

Only azines with both 2- and 6-positions free versus at least one nitrogen atom in the azine ring produced high-colored compounds when treated with TCMB, probably due to steric hindrance effect. For the same reasons, there was no color reaction with quinoline, isoquinoline, quinine, and 1,10-phenantroline as well. 4-Hydroxypyridine did not react because the tautomeric pyridone was probably formed in solution [5].

### 2.2. Azoles and Benzazoles

In the same manner, the chromogenic reactions of (**1**) with five-membered heteroaromatic rings containing 1 to 4 nitrogen atoms without [6] or with [7] condensed benzene ring and their application for detection of these compounds on TLC plates were also described.

The following heteroaromatic nitrogen containing compounds with 5-member ring gave no chromogenic reaction after spraying with 1% TCMB acetone solution on TLC plates: 1-methyl pyrrole, pyrazole, 1- and 3-methyl pyrazole, imidazole, 4-methyl and 4-nitroimidazole, 1-methyl-(1*H*)-1,2,3-triazole, 2-methyl-(2*H*)-1,2,3-triazole, 1,2,4-triazole, 1-methyl- and 3-nitro-(1*H*)-1,2,4-triazole, (1*H*)-1,2,4-triazole-3-thiol, 1-methyl-(1*H*)- and 2-methyl-(2-*H*)-tetrazole, (1*H*)-1,2,3,4-tetrazole-5thiol [6], oxazole, isoxazole, 4-methyloxazole, thiazole, isothiazole, 2-methylbenzothiazole, 2-mercaptomethylbenzothiazole, 1methylindole, benzimidazole, benzotriazole, 1-methylbenzotriazole, and 2-methylbenzotriazole [7].

It was found that probably two different reaction mechanisms took place [8].

- For azoles without three vicinal nitrogen atoms in the heteroaromatic ring and/or a weakly acid NH group, a chromogenic reaction I [6] analogous to that described for the azines [5] seems to occur. The necessary conditions for this reaction to occur are that (i) both *α*-positions should be unsubstituted with respect to the basic tertiary nitrogen atom –N= and (ii) the labile hydrogen atom at the weakly acidic secondary nitrogen NH should be protected by substitution with an alkyl, for example, in the imidazole moiety, 1-methylimidazole **22**, or 1ethylimidazole **23**.
- For the compounds with a more acidic hydrogen in the NH group, such as pyrrole **21**, or with three or more contiguous nitrogen atoms in the azole ring, as in 1,2,3-triazole **24, **4-methyl-(4*H*)-1,2,4-triazole **25 **and tetrazole **26**, the previous reaction did not take place or could be a side reaction, but another mechanism—**chromogenic reaction II**—might be observed. Pyrrole **21 **and 1,2,3-triazole **24** unexpectedly reacted quickly even at room temperature, whereas 1-methylpyrrole and both 1- and 2-methyl-1,2,3-triazoles did not form any intensively colored product with TCMB. In the reaction of TCMB with an excess of 1,2,3-triazole **25 **as a solvent, the formation of a brown product and evolution of nitrogen were observed. No volatile products except unreacted starting materials were found. Probably the decomposition of triazole **25** and TCMB occurs with evolution of nitrogen and the formation of unidentified products [8].
- The same type of mechanism of **chromogenic reaction II** was observed in reactions of TCMB **1** with nitrogen containing heteroaromatic compounds with the condensed benzene ring [7]. It was found the high-color reactions occurred with *benzo*- homologues of 1,2,3-triazole and pyrrole—benzotriazole **27** and indole **28**, as well as with benzothiazole **29** and 2-mercaptobenzothiazole **30**; most probably the second mechanism took place with elimination of acetylene, nitrogen, carbon monosulfide, and disulfide, respectively [8] (see Table 2).

### 2.3. Structure of LI Complex and Mechanisms of Chromogenic Reactions

**Figure 1:**
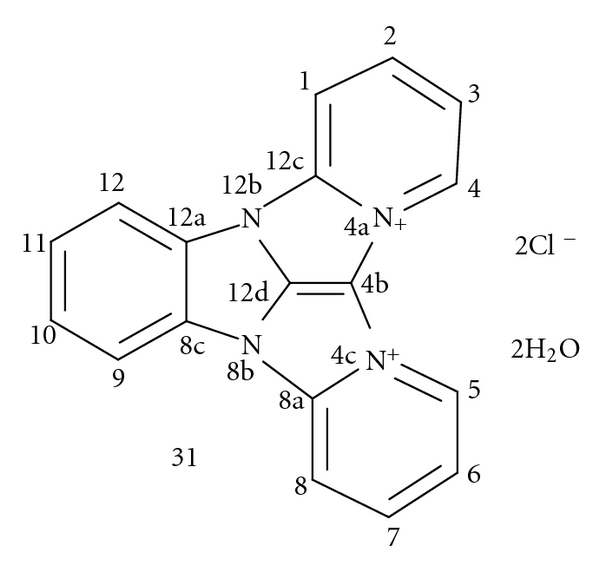
The structure of compound **31**, the main component of the LI Complex.

We have identified the structure of the main component **31** of the **LI Complex**, the amaranth product of the reaction of TCMB **1** with pyridine **2**. It was a complex mixture of highly polar, intensively colored, and unstable products. The crude LI Complex was purified by preparative TLC on precoated polyamide aluminum sheets. The main amaranth compound **31** was characterized using the following analytical and spectral evidence: elemental analysis, EI MS, HR EI MS, FD MS, ^1^H NMR, IR, UV-VIS [9], and ^13^C NMR [8]. The deuterium labelled octadeuterated compound **31-** **d**_8_ was also obtained when the chromogenic reaction was carried out with pyridine-*d*_5_. The details are described in the paper [9]. It was found the most probable structure of the compound **31** was 4a,4c,8b,12b-tetraazadibenzo [*a*,*f*] indano [1,2,3-*cd*] pentalene-4a,4c-diinium dichloride dihydrate (IUPAC) or 8b,12b-diaza-4a,4c-diazoniadibenzo [2,3 : 4,5] pentaleno [1,6-*ab*]indene dichloride dihydrate (CAS); CAS RN [*127024-68-4*] (Figure 1).

**Figure 2:**
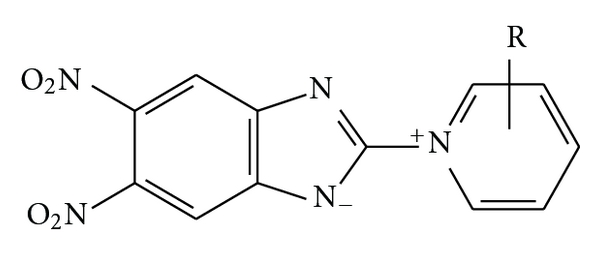
Quaternization of 5,6-dinitro-2chlorobenzimidazole **32** by pyridine derivatives producing betaines [10].

In the literature, there are only few references concerning this type of reaction. Postovskii et al. [10] described quaternization of 5,6-dinitro-2-chlorobenzimidazole **32** in boiling by pyridine (**2**) and 3- or 4-picolines receiving yellow betaines after elimination of hydrochloric acid (Figure 2). However, the reaction conditions were substantially more drastic (boiling heterocyclic amine, 1 h) than for TCMB (room temperature, 2 min). Moreover, the chlorine atom in **32 **was much more mobile due to presence of nitro groups in the benzene ring. Similarly to examined reaction of TCMB and picolines, 2-picoline did not react [8, 10].

**Figure 3:**
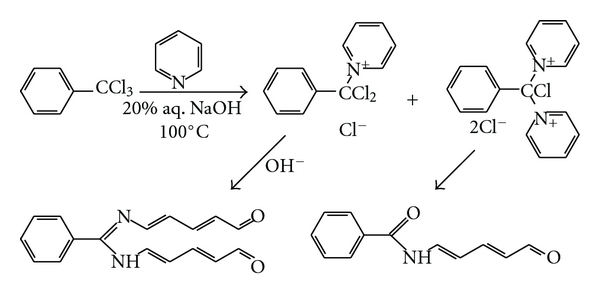
The Fujiwara reaction mechanism [11, 12].

Kyoyetsuro Fujiwara was a Japanese POW in Germany during the WW I, and in 1916 the Fujiwara reaction [11] taking place between *α*,*α*,*α*-trichlorotoluene **33** and pyridine **2** in rather drastic conditions (20% aqueous sodium hydroxide at 100°C) was first described. Two high-colored products, yellow and red, are formed (Figure 3). Reaction was extremely sensitive. The Fujiwara reaction application was reviewed [7, 8, 11, 12].

However, the conditions of the reaction of TCMB with pyridine were very mild and its mechanism and structures of obtained products were different.

Thus, it was found that probably **two** different reaction mechanisms took place [8]:

- with electron deficient basic heterocyclic compounds as azines, for example, pyridine (type **A**);
- with heteroaromatic compounds containing acid NH group as azoles, for example, a benzazole (type **B**).

**Figure 4:**
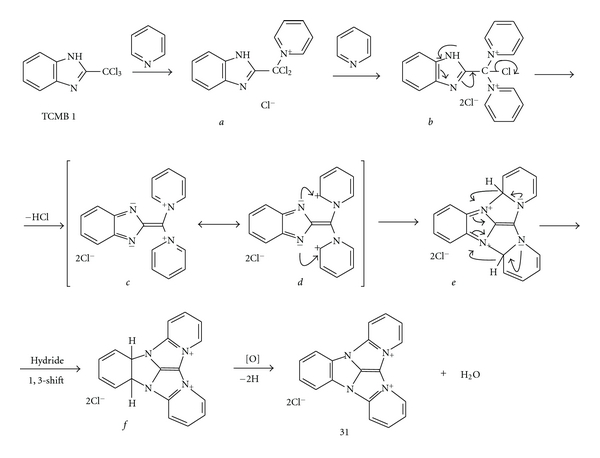
Mechanism of chromogenic reaction I (**A**) of TCMB **1 **and pyridine **2** [8].

**(A)** For the electron deficient basic heteroaromatic compounds without acid N-H group or when this group is protected, and when at least one tertiary nitrogen =N–atom without substituents at two adjacent carbon atoms is present in the ring, such as pyridine, diazines (pyridazine, pyrimidine and pyrazine), *N*-substituted imidazole, and 4-substituted 1,2,4-triazole derivatives, the following mechanism of the compound **31** formation seems to be the most probable (mechanism **A**, with pyridine **2** as an exemplary heterocyclic compound, Figure 4).

**Figure 5:**
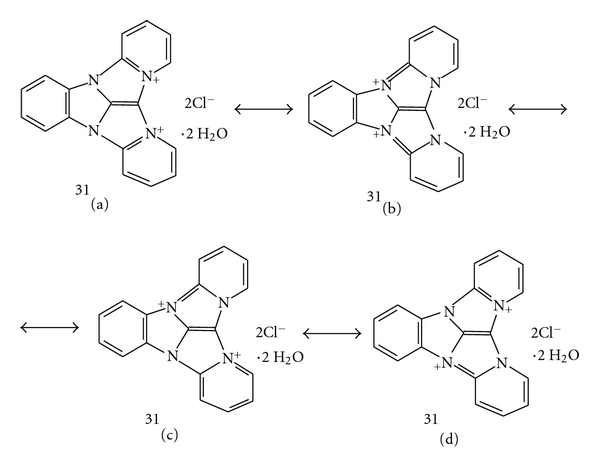
Mesomeric structures of the compound **31**, the main component of **LI Complex **[8].

The obtained polycyclic diinium compound **31** is stabilized by the intramolecular charge delocalization (Figure 5).

However, in the ^13^C NMR spectrum 18 separated carbon signals can be observed. It shows that the molecule is twisted, the carbons in both pyridinium rings are not equivalent, and the asymmetric resonance hybrids **31c** and **31d** have a large contribution in the real molecule structure. Additionally, the positive charges localization at two relatively closed nitrogen atoms, as in the structures **31a** and **31b,** seems to be less favorable than in the structures **31c** and **31d**, where the distance between charged atoms is longer.

**Figure 6:**
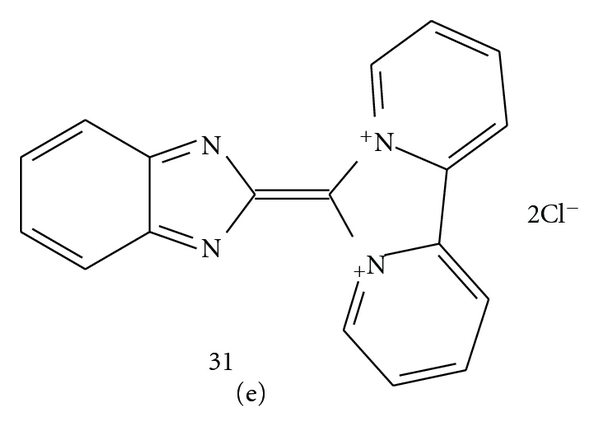
**31e**, the alternative structure of the compound **31.**

Since there is a partial positive charge at the *α*- and *γ*-carbon atoms in the pyridinium cation *c*, the nucleophillic attack of the electron pairs in its di-imine mesomeric form *d* at an *α*-carbon in each pyridinium ring and the dihydropyridine intermediate *e* formation was found much more probable than the formation of a new C–C bond and formation of the compound **31e (** Figure 6).

The reaction product analysis and the ionic chlorine presence [9] let to exclude the possibilities of pyridine ring cleavage and the conjugated polyenes formation in Fujiwara reaction [11, 12] as well as betaine products of the reactions of 5,6-dinitro-2-chlorobenzimidazole (**32**) with pyridine derivatives [9]. The reaction precondition is the absence of any substituent at any carbon atom adjacent to the nitrogen. However, the second adjacent *α*-position must be also free, perhaps due to steric reasons, most probably during the intermediates *a* and *b* formation. The dehydrogenation of intermediate *e *was necessary for the compound **31** formation. The most probable seems to be rearomatization of dihydropyridine *e* by double 1,3-hydride shift giving the intermediate *f*. In the literature, it was not found any similar examples of intramolecular 1,3-hydride transfer in such heterocyclic systems, but hydride ion transfer is proved to be typical for reductions by dihydropyridines [13]. The last step was aromatization of *f* into the final product **31**. It was observed that the dehydrogenation of *f *is due mainly to oxidation by atmospheric oxygen, since in the atmosphere of an inert gas, such as argon, the reaction of TCMB with pyridine occurs much more slowly. However, other oxidation mechanisms should not be excluded, for example, the oxidative aromatization as it was found in last stage of the Skraup quinoline synthesis [14].

**Figure 7:**
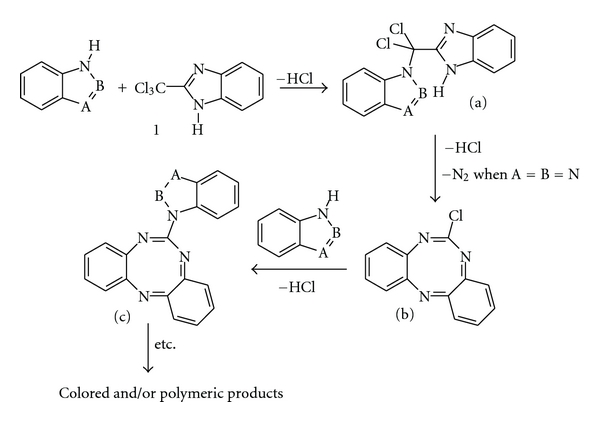
Mechanism of chromogenic reaction II **(B)** of TCMB (**1**) and a benzazole.

**(B)** For the compounds with a more acidic hydrogen in the N-H group, such as pyrrole (**21**) and indole (**28**) or with three or more contiguous nitrogen atoms in the azole ring as benzotriazole (**27**), 1,2,3-triazole (**24**), and tetrazole (**26**), as well as for benzothiazole **29** and 2mercaptobenzothiazole (**30**), another reaction mechanism may be observed (mechanism **B**, with a benzazole as an exemplary heterocyclic compound, Figure 7).

The necessary reaction condition is the presence of acidic secondary NH group, which reacts with TCMB. In this case the quaternization does not take place, but the substitution followed by the hydrogen chloride abstraction seems to occur, and this acid is neutralized by the amine excess. However, the heterocyclic compound should not be a too weak N–H acid; therefore this reaction was not observed with pyrazole, imidazole, 1,2,4-triazole, or benzimidazole.

In the next step, the reaction is favored also by elimination of a stable and volatile molecule leaving the reaction medium; in the cases of 1,2,3-triazole (**24**), tetrazole (**26**), and benzotriazole (**27**), the nitrogen elimination was observed. 2-Mercaptobenzothiazole (**30**) can eliminate the carbon disulfide molecule from its 2-benzothiazolethione tautomeric form (**30a**); therefore this compound reacted easily with TCMB, whereas 2-methylbenzothiazole and 2-mercaptomethylbenzothiazole, which do not form any thione tautomers, do not react with TCMB [7]. In next steps, abstraction of another molecule of HCl and re-cyclization may occur to a structure like *b*, high-colored due to several conjugated double bonds. Then, another hydrogen chloride molecule may be eliminated with one more condensation and formation of structure *c* or similar, and so forth.

The structure of finally obtained dark, unstable, nonvolatile, and probably complex polymeric products of the reactions of the **B** type seems to be difficult to identify.

## 3. Examples of Application of the TCMB Method for Determination of Organic Compounds on TLC Plates

### 3.1. Detection of Aromatic *o*-Diamines [15]

**Figure 8:**
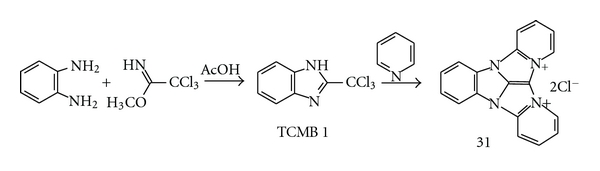
Detection of *o*-phenylenediamines on TLC plates.

The classical method of the 2-substituted benzimidazoles synthesis is a condensation of *o-*phenylenediamine and a carboxylic acid with a basic catalyst. However, trichloroacetic acid does not react with *o*-phenylenediamine [1]. That is why in its original preparation, TCMB was synthesized in condensation of methyl trichloroacetimidate (**33**)[16] and *o*-phenylenediamine (**34**) in acetic acid at RT [1]. The prechromatographic reaction of aromatic *o*-diamines and commercial methyl trichloroacetimidate (**33**) with TCMB formation, followed by TLC and visualization in the same dyeing reaction (10–15 min in pyridine vapors at RT), was applied for detection of *o*-phenylenediamine (**35**) and some of its derivatives (Figure 8, Table 3)[15].

Some compounds gave no color reaction using this method of visualisation:

- *ortho-* and *peri*-naphthylenediamines and 2,3-diaminophenazine, probably for steric reasons;
- *m-* and *p-*phenylenediamines and aliphatic 1,2-diamines—since the aromatic benzimidazole ring cannot be formed,
- *N-*methyl-*o*-phenylenediamine, which probably forms a corresponding 1-methyl-2-trichloromethylbenzimidazole in reaction with methyl trichloroacetimidate, but the heterocyclic obtained does not react subsequently with pyridine and the high-colored LI Complex does not form.

### 3.2. Determination of Heteroaromatic Pesticides on TLC Plates Using Scanning Spectrodensito-Metry [17, 18]

The method of spraying with 1% acetone solution of TCMB as a chromogenic agent was applied for visualization for 1 min at 150°C of selected pyridine, pyrimidine, pyrrole, indole, and conazole pesticides on TLC plates. Both chromogenic reactions I (for 6-member ring heterocycles) and II (for 5-member rings) were observed. The detection limit (LOD) was evaluated visually [17] for pesticides **39–46**. The quantitation of the thin-layer chromatograms of the other pesticides **47–51** was performed using a scanning spectrodensitometer (Camag TLC Scanner 3) at selected wavelengths 430, 460, and 498 nm; scanning speed was 10 mm/s [18]. The relative standard deviation (RSD) values were calculated as level of experimental error using the Student *t*-distribution at a significance level *α* = 0.05, and the relative error was less than 15% (Table 4) [18].

The LODs were calculated as the average from three to five experiments, as the interpolated amount of each compound corresponding to the densitometric peak height equals the tripled average noise level in the detected compound region of the densitogram [18].

Such calculations could not be performed for chromatograms on developed TLC plates where there are no peaks but only spots, and only the smallest still visible spot may be considered as the LOD evaluated visually in this semiquantitative method. So, the full validation of the method for precision, accuracy, reproducibility, and limit of quantification (LOQ) was not performed. The linearity range was not determined as well. However, if the spot area *A* would be measured using a planimeter, and the amount of determined compound would be equal *c*, the square root of *A* should be proportional to logarithm of *c*:

(1) $$\sqrt{A}=k\cdot \log c+b,$$

where *k* and *b* are constants for a given chromatogram.

It was experimentally checked and calculated for fenpiclonil **49** that this equation was really linear, and coefficient of determination *R* ^2^  equals  0.996.

### 3.3. Dosimeters for the Nicotine Presence in Air Using the Chromogenic Reaction between TCNB and Nicotine [19]

The possibilities of application of the chromogenic reaction with TCMB for detection of the residual amounts of nicotine in air were studied. LOD of nicotine in this reaction on TLC plates is 20 ng (spot color: yellow).

Passive and active dosimeters were tested. The **passive dosimeter**s were made of glass tubes, 4 cm length × 0.4 cm i.d. filled with 5 or 10% TCMB adsorbed on Merck Silicagel 0.2–0.5 mesh or 50–120 mesh. Both ends of the tube were protected with a loose plug made of glass cotton. The passive dosimeters were exposed in various locations where the cigarettes were smoked intensively (smoking room), moderately (designated areas), or not at all (laboratories). In some tubes, the testing phase got yellowish. Unfortunately, TCMB turned out very sensitive on light and air pollutants and got decomposed to yellow products with color similar to reaction product with nicotine. That is why there was found no essential correlation between presence of tobacco smoke in the examined area and intensity of the color in the testing tube.

Next, the **active dosimeters** were tested. They had got a similar construction as the passive ones; only the glass tube was longer (10 cm) and contained 1 to 2 layers of 5–10% TCMB on Silica. The measurements were carried out by blowing the smoke from a lighted cigarettes with changeable velocity (0.5–2 cigarette/min) using just a water aspirator pump. The experiment was carried out with cigarettes of different manufacturers. A compact filter with cellulose wool was indispensable, since the cigarette tar dyed strongly all the tube and the reading was impossible. It was found that independently of the cigarette mark and time of “smoking” (0.5 to 2 min), after smoking about 1/4 of the cigarette the TCMB on silica in the dosimeter tube changes the color and got yellow. Checking the presence of nicotine in the air respired by a smoking person was also possible. The silica gel with larger particle size was found to be better as the dosimeter filling because it enables better air circulation.

 Unfortunately, to develop the dosimeter readings, it was necessary to heat the dosimeter up to 150°C for 1 min or to 100°C for 10 min, or else leave it at RT (ca. 20°C) for 30 min. It can seriously hamper the practical application of such a designed active smoking dosimeter.

## 4. Application of TCMB and Pyridine Reaction Product (LI Complex) as a New Chromogenic Reagent for Analysis of Inorganic Compounds [20]

LI Complex, a deep amaranth, polar, and well soluble in water compound (intensively red in water solutions) with a structure of 4a, 4c, 8b, 12b-tetraazadibenzo [*a*,*f*] indano [1,2,3-*cd*] pentalene-4a,4c-diinium dichloride dihydrate (**31**), was found to change color in presence of some inorganic cations, affording complex compounds. Thus, it can be used as a chromogenic agent in analysis of inorganic salts as well. The highly colored complexes formation in presence of several inorganic anions were observed as well; it was obvious since LI Complex is a dication. In most cases, a high-colored complexes formation in presence of several inorganic anions was observed as well; it was obvious since LI Complex is a dication. In most cases, a high-colored precipitate formation occurred. The details are presented in Table 5.

An analytical importance can especially have reactions of LI Complex with silver (**I**) cation (purple coloration), mercury (**II**) dication (brown-red coloration), and lead (**II**) dication (red coloration, but a copious orange precipitate). Thus, this chromogenic agent can be applied as a complexometric indicator in solutions as well as an adsorption indicator (colored precipitates formation). Some anions (e.g., pyrophosphates, periodates, or dichromates) may also produce high-colored products with LI Complex.

 Color change after addition of water solutions of Na_2_CO_3_, NH_4_OH, and HCl of various pH allows to suppose that LI Complex may also be used as a sensitive indicator in acid-base titration. Indeed, we have found that this method may have the following well-established titration parameters.

- The aqueous LI-Complex should be used at concentrations 3 to10 g/L water.
- The colors of indicator were as follows: yellow in acidic solutions and brown-purple in the basic ones, and the intermediate colors were orange-pink and red.
- Since LI Complex is a dication, there are two transition points during the titration.
- The color transition points were found:
  - at weakly acidic pH region (4 to 4.5, from yellow into yellow-pink),
  - at neutral region (pH 6.5 to 7), from orange-pink into red.

## 5. Conclusions

LI Complex is the main product of reaction of 2-trichloromethylbenzimidazole (TCMB, **1**) with pyridine, the simplest and the most representative example of series of reactions described in this paper. In this reaction, a complex and difficult to identify high-colored mixture of polar compounds is formed, and the reaction seems to be very specific and regioselective. The structure of the LI Complex main component was established as 4a,4c,8b,12b-tetraazadibenzo[*a*,*f*] indano[1,2,3-*cd*] pentalene-4a,4c-diinium dichloride dihydrate (**31**).

This reaction was subsequently used to develop a package of analytical methods in both organic and inorganic chemistry. As its main application, TCMB showed to be a sensitive and specific chromogenic agent to visualize and determine some organic compounds on TLC plates: azines, azoles, benzazoles, *o-*phenylenediamines, and heteroaromatic pesticides (also with scanning spectrodensitometry of developed TLC plates). The mechanisms of two different chromogenic reactions of TCMB with azines and azoles were proposed and proved. The attempts to apply one of the aforementioned methods to develop passive and active nicotine dosimeters were also discussed. Finally, the applications of the LI Complex for analysis of inorganic cations and another application of this reagent as an indicator in acid-base titration were presented.

Nonetheless, the bunch of methods developed about the triangle TCMB—pyridine—LI Complex has its limitations. TCMB may be used as a chromogenic agent mainly in TLC, which is often considered to be only a semiquantitative method, not frequently used by analytical chemists (but it is commonly used by synthetic organic chemists for control the reaction progress on TLC plates, and they are also the target of this paper). Moreover, the color reactions in most cases should be accelerated at high temperature, usually at 100–150°C for several minutes, and it makes the analytical procedures more complicated.

However, there are not many analytical methods as universal as the ones described previously. These methods were quoted in many publications, for instance, [21–30]. Moreover, TCMB may be easily synthesized from commercially available methyl trichloroacetimidate and *o*-phenylenediamine [1].

## Figures and Tables

**Table 1:** Colors and detection limits of the examined azines visualized with TCMB on TLC plates [5].

Compound no.	Azine (common name)	Color (for 1000 ng in spot)	Detection Limit LOD (ng)
2	Pyridine	Amaranth	50
3	Pyridazine	Red	20
4	Pyrimidine	Gray-green	100
5	Pyrazine	Yellow	100
6	3-Metylpyridine (*β*-picoline)	Amaranth-brown	20
7	4-Metylpyridine (*γ*-picoline)	Ochre-yellow	20
8	4,4′-Bipyridil	Orange	10
9	3-Formylpyridine	Ochre-yellow	20
10	4-Formylpyridine	Ochre-yellow	20
11	4-Cyanopyridine	Orange	20
12	4-Aminopyridine	Yellow	50
13	Nicotinic acid (Vitamin PP)	Yellow-green	10
14	Isonicotinic acid	Yellow-green	10
15	Methyl nicotinate	Yellow	20
16	Nicotinamide (Vitamin PP)	Yellow	20
17	*N*-(Hydroxymethylnicotinamide) (Cholamid)	Ochre-yellow	20
18	Isonicotinohydrazide (Isoniazid)	Orange	20
19	Nicotine	Yellow	20
20	2-Pyrazinecarboxamide	Orange	20

**Table 2:** Colors and detection limits of the examined azoles [6] and benzazoles [7] visualized with TCMB on TLC plates.

Compound no.	Azole	Color (for 10 *μ*g in spot)	Detection limit (LOD, ng)	Mechanism of chromogenic reaction
**21**	Pyrrole	Brown	500	II
** 22**	1-Methylimidazole	Brown-yellow	10 000	I
**23**	1-Ethylimidazole	Yellow	5000	I
**24**	1,2,3-Triazole	Brown	50	II
**25**	4-Methyl-(4*H*)-1,2,4-triazole	Yellow	1000	II
**26**	Tetrazole	Khaki	1000	II
**27**	Benzotriazole	Yellow	300	II
**28**	Indole	Amaranth	100	II
**29**	Benzothiazole	Grey	1000	II
**30**	2-Mercaptobenzothiazole	Brown	500	II

**Table 3:** Colors and detection limits of the examined *o*-phenylenediamines on TLC plates [15].

Compound no.	Azole	Color (for 1000 ng in spot)	Detection limit (LOD, ng)
**36**	*o*-phenylenediamine	Amaranth-brown	100
**37**	3,4-diaminotoluene	Brown	100
**38**	4-nitro-*o*-phenylenediamine	Brown	80

**Table 4:** Colors and low detection limits (LOD) of the examined pesticides on TLC plates [17, 18].

Cmpd no.	Common name	Color (for 10 *μ*g in spot) (Wavelength, nm)	Detection limit (LOD, ng)	Chromogenic reaction/Evaluation	Type of pesticidal activity
39	Nicotine	Yellow	20 ± 6	I/visually	Insecticide
40	Pyrifenox	Brown	25 ± 4	I/visually	Fungicide
41	Flurprimidol	Ochre-orange	50 ± 13	I/visually	Plant growth regulator
42	Fenarimol	Yellow	100 ± 16	I/visually	Fungicide
43	Prochloraz	Orange	2000	II/visually	Fungicide
44	Bitertanol	Yellow	1000 ± 200	II/visually	Fungicide
45	Paclobutrazol	Brownish	10 000	II/visually	Fungicide
46	Flutriafol	Orange	1800 ± 200	II/visually	Fungicide
47	Indol-3-ylacetic acid, IAA	Brown,/460/	75 ± 10	II/densitometry	Plant hormone
48	Indol-3-ylbutyric acid, IBA	Brown-yellow,/460/	650 ± 100	II/densitometry	Plant growth regulator
49	Fenpiclonil	Brown-red,/498/	250 ± 40	II/densitometry	Fungicide
50	Ketoconazole	Ochre,/460/	120 ± 15	II/densitometry	Fungicide
51	Fluconazole	Yellow,/430/	490 ± 60	II/densitometry	Fungicide

**Table 5:** Reactions of Complex LI (*c* = 10 g/L in water) with inorganic compounds (*c* = 100 g/L in water).

No.	Compound	Color of solution		Precipitate	After 24 h
		Before LI Complex addition	After LI Complex addition		
1	**Cu**SO_4_·5 H_2_O	Blue	Dark-red	None	None
2	**Ag**NO_3_	Colorless	Purple	Purple	Purple precipitate, solution over the precipitate– yellow-green
3	**Hg**(NO_3_)_2_·1/2 H_2_O	Colorless	Brown-red	Brown-red	Brown-red precipitate
4	**O=V**SO_4_	Blue	Terra-Cotta	None	Green solution
5	**Cr**(NO_3_)_3_	Navy blue	Red, turbid	Orange	Orange precipitate, solution over the precipitate– reddish
6	**Co**(NO_3_)_2_·6 H_2_O	Pink	Orange	Orange	No change
7	**Pb**(NO_3_)_2_	Colorless	Red, turbid	Orange	Orange precipitate, solution over the precipitate– dark yellow
8	Na_4_ **P_2_O_7_**·10 H_2_O	Colorless	Navy blue	Navy blue	No change
9	Na**IO_4_**	Colorless	Bright yellow	Red	No change
10	K_2_ **Cr_2_O_7_**	Orange	Dark red	Dark red	No change
11	Na_2_CO_3_ (pH 10)	Colorless	Navy blue-green	Slightly turbid	Navy blue precipitate
12	NH_4_OH (pH 9)	Colorless	Brown-purple	Black-brown	No change
13	HCl (pH 1)	Colorless	Yellow	Pale orange	No change
